# Transmission, relatedness, and the evolution of cooperative symbionts

**DOI:** 10.1111/jeb.13505

**Published:** 2019-07-28

**Authors:** Asher Leeks, Miguel dos Santos, Stuart A. West

**Affiliations:** ^1^ Department of Zoology University of Oxford Oxford UK; ^2^ Department of Social Psychology and Social Neuroscience University of Bern Bern UK

**Keywords:** cooperation, evolutionary branching, kin selection, mutualism, symbiosis

## Abstract

Cooperative interactions between species, termed mutualisms, play a key role in shaping natural ecosystems, economically important agricultural systems, and in influencing human health. Across different mutualisms, there is significant variation in the benefit that hosts receive from their symbionts. Empirical data suggest that transmission mode can help explain this variation: vertical transmission, where symbionts infect their host's offspring, leads to symbionts that provide greater benefits to their hosts than horizontal transmission, where symbionts leave their host and infect other hosts in the population. However, two different theoretical explanations have been given for this pattern: firstly, vertical transmission aligns the fitness interests of hosts and their symbionts; secondly, vertical transmission leads to increased relatedness between symbionts sharing a host, favouring cooperation between symbionts. We used a combination of analytical models and dynamic simulations to tease these factors apart, in order to compare their separate influences and see how they interact. We found that relatedness between symbionts sharing a host, rather than transmission mode per se, was the most important factor driving symbiont cooperation. Transmission mode mattered mainly because it determined relatedness. We also found evolutionary branching throughout much of our simulation, suggesting that a combination of transmission mode and multiplicity of infections could lead to the stable coexistence of different symbiont strategies.

## Graphical abstract

Vertical transmission can favour symbionts that cooperate more with their hosts for two reasons: by aligning the fitness interests of hosts and symbionts; and by increasing the relatedness between symbionts. Using theoretical models, we contrasted both mechanisms, and found that increasing relatedness played the bigger role.

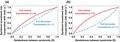

## 1 INTRODUCTION

There is considerable variation in the benefit that hosts gain from their symbionts. In some cases, hosts are completely dependent upon their symbionts. For example, aphids cannot survive or reproduce without *Buchnera* symbionts, which provide essential amino acids (Buchner, 1965; Douglas, 1998). In other cases, symbionts appear to provide relatively minor benefits. For example, the removal of *Chlorella* symbionts from *Paramecium bursaria* leads to just a reduction in growth rate, and only under certain conditions (Karakashian, 1963; Lowe et al., 2016). Empirical studies have suggested that the way in which symbionts are transmitted between hosts plays an important role in explaining this variation (Bull et al., 1991; Bull & Molineux, 1992; Herre, 1995; Messenger et al., 1999; Sachs & Wilcox, 2006; Fisher et al., 2017). Specifically, that vertical transmission, where hosts transmit symbionts to their offspring, selects for more cooperative symbionts than horizontal transmission, where symbionts can leave their host and be transmitted to other individuals in the population. Symbionts which are more cooperative could in turn provide greater benefits to their hosts, by investing more of their resources into functions which benefit their hosts or by refraining from overexploiting their hosts’ resources (Frank, 1994, 1996).

Two different mechanisms have been given for why the mode of symbiont transmission matters (Frank, 1996). One mechanism is that if symbiont offspring are likely to be transmitted to host offspring, then symbionts benefit when the host has more offspring (Ewald, 1987; Yamamura, 1993, 1996; Ferdy & Godelle, 2005). In this “transmission” scenario, it is vertical transmission per se that selects for higher levels of symbiont cooperation, through aligning the fitness interests of hosts and symbionts—vertical transmission makes symbionts more dependent upon their hosts. The other mechanism is that the transmission route determines the genetic diversity or relatedness between the symbionts and that this determines selection for cooperation (Hamilton, 1964; Frank, 1994, 1996; Herre et al., 1999; West et al., 2002; Foster & Wenseleers, 2006). Greater horizontal transmission will lead to a lower relatedness between symbionts. As relatedness between symbionts goes down, this can favour symbionts who avoided the cost of helping their hosts, but could still benefit from the benefits provided to the hosts by other symbionts. In this “relatedness” scenario, transmission mode matters, but it does so through its influence on relatedness—vertical transmission reduces conflict between symbionts.

Both of these mechanisms, “transmission” and “relatedness”, could operate, and both their relative importance and the extent to which one influences the other remain unclear. The empirical observation that vertically transmitted symbionts provide greater benefits to their hosts could be explained by either mechanism, or by both acting simultaneously. Theoretical studies tend to make simplifying assumptions that allow them to focus on just one of these mechanisms (Frank, 1996). For example, some of the studies that emphasize transmission mode assume that hosts can only be infected by one strain of symbiont at a time, ignoring the possibility for conflict between symbionts within a host (Yamamura, 1993, 1996). Similarly, models that examine the influence of variable relatedness do not usually explicitly model horizontal and vertical transmission (Frank, 1994, 2010). In nature, both mechanisms are likely to occur, and we have a poor understanding of the consequences. For example, would they have distinct and different influences, or would they interact; would one drive the other, or would one tend to dominate?

We use a three‐pronged theoretical approach to investigate how these different mechanisms could interact, and their relative importance (Frank, 1996). We first build an analytical model of a specified symbiont life cycle in which we can tease apart the separate causal influences of relatedness and transmission mode. This allows us to test which mechanism plays the larger causal role in the evolution of cooperation. Then, by expressing relatedness in terms of symbiont transmission mode and bottlenecking between symbiont generations (“closing” the model), we allow transmission mode to influence relatedness. This allows us to partition the influence of transmission mode per se, and via its effect on relatedness (Cooper et al., 2018). Finally, we test the robustness of our conclusions with an individual‐based simulation. This simulation allows us to relax several assumptions, including that mutations are of small size, and that the trait value for cooperation does not influence relatedness. Our simulation also allows us to investigate whether evolutionary branching can occur, as has been observed in the early stages of experimentally evolved mutualisms (Harcombe et al., 2018).

## 2 MODELS AND RESULTS

### 2.1 Assumptions and model life cycle

We assume a mutualism in which symbionts live inside hosts and potentially provide them with some benefit. We assume that the symbionts cannot survive long enough to reproduce outside the hosts, and so they are obligately dependent on the hosts. We assume that there is an infinite population of hosts with nonoverlapping generations and that there is no host population structure.

We assume that the cooperative symbiont trait *x* denotes the amount of resources contributed towards a service which benefits the host, but which does not directly benefit the symbiont. For example, this trait could be the production of a key nutrient that the host needs. We assume that hosts with more cooperative symbionts are more likely to survive to reproductive maturity and are more likely to produce more offspring after reaching reproductive maturity. Therefore, we assume that symbiont cooperation can benefit both host survival and host fecundity, according to the functions *s*(*x_g_*) and *f*(*x_g_*), respectively, where *x_g_* refers to the mean investment into cooperation of all of the symbionts inside a focal host. We use mean, and not total, symbiont investment into cooperation, for the sake of simplicity, and to be consistent with previous work (Frank, 1994, 1996). We also assume that this trait is costly to the symbiont, by assuming that a focal symbiont's growth rate inside a host depends negatively on its investment into cooperation, according to the expression $\frac{1-x_{i}}{1-x_{g}}$, where *x*_i_ is a focal symbiont's investment into cooperation.

We assume that a symbiont can potentially transmit offspring to future generations via two routes, vertical or horizontal: vertical transmission occurs when a symbiont's offspring remain in their host and are passed on to the host's offspring; horizontal transmission is when a symbiont's offspring can infect the offspring of any host in the population. We assume that increased host survival increases the transmission opportunities for horizontally transmitting symbionts, and so we weight the horizontal component of symbiont fitness by a focal symbiont's host's relative survival, $\frac{s\left(x_{g}\right)}{s\left(\overset{¯}{x}\right)}$, where $\overset{¯}{x}$ is the mean level of symbiont cooperation in the population as a whole. We assume that both host survival and host fecundity per unit time increase the transmission of vertically transmitting symbionts, and so we weight the vertical component of symbiont fitness by $\frac{s\left(x_{g}\right)}{s\left(\overset{¯}{x}\right)}\frac{f\left(x_{g}\right)}{f\left(\overset{¯}{x}\right)}$.

Finally, we use a parameter *λ* to capture the relative likelihood of horizontal (*λ*) compared to vertical (1‐ *λ*) transmission. *λ* could be influenced by a number of different biological factors, including if hosts are more likely to reject symbionts from one route than the other, or if one mode of transmission involves higher symbiont mortality. The fitness of a focal symbiont is then:

(1) $$W=\left(1-\lambda \right)\frac{\left(1-x_{i}\right)}{\left(1-x_{g}\right)}\frac{s\left(x_{g}\right)}{s\left(\overset{¯}{x}\right)}\frac{f\left(x_{g}\right)}{f\left(\overset{¯}{x}\right)}+\lambda \frac{\left(1-x_{i}\right)}{\left(1-x_{g}\right)}\frac{s\left(x_{g}\right)}{s\left(\overset{¯}{x}\right)}.$$

This fitness equation sets up a trade‐off similar to other models of cooperative traits (Frank, 1994, 2010). Figures were produced using Wolfram Mathematica 11.3 (Harrower & Brewer, 2003; Wang, 2016).

### 2.2 Equilibrium analysis

We are interested in the level of investment into cooperation (*x* ^*^) which, if adopted by all symbionts in the population, could not be beaten by any alternative value of *x*, which is termed an evolutionarily stable strategy (ESS). We used a neighbour‐modulated fitness approach to obtain the inclusive fitness effect, *∆*_IF_, of small changes in the trait value for cooperation on the inclusive fitness of a focal individual, assuming the limit of weak selection (Taylor & Frank, 1996):

(2a) $$\Delta \text{IF}=\frac{\partial W}{\partial x_{i}}+R\frac{\partial W}{\partial x_{g}}$$

We solved △IF = 0 for x*, evaluating all derivatives at $x_{i}=x_{g}=\overset{¯}{x}=x^{*}$ (Maynard Smith & Price, 1973). To allow for a wide range of relationships between symbiont cooperation and host survival or fecundity, we assume that $s\left(x_{g}\right)=x_{g}^{s}$ and $f\left(x_{g}\right)=x_{g}^{f}$, where *s* > 0 and *f* > 0, and so arrive at:

(2b) $$\Delta {\text{IF}}_{x_{i}=x_{g}=\overset{¯}{x}=x^{*}}=-\frac{1}{1-x^{*}}+R\left[\frac{s+f\left(1-\lambda \right)}{x^{*}}+\frac{1}{1-x^{*}}\right]$$

where higher values of *f* or *s* indicate that host fecundity or survival respectively increases more quickly with symbiont cooperation, and *R* is the whole‐group relatedness coefficient (Taylor & Frank, 1996; Pepper, 2000).

Equation 2b allows us to see the different effects of changes in cooperation (x*) on the inclusive fitness of a focal individual. The first term in Equation 2b is the cost of cooperation (x*), which reflects reduced symbiont competitiveness within a host. The second term in Equation 2b is the benefit of cooperation that goes to the other symbionts sharing the focal symbiont's host, weighted by the genetic relatedness between the focal symbiont and its neighbours (*R*). This benefit stems from the fact that more cooperative (higher x*) groups of symbionts will have hosts that live longer (in a way that scales with *s*) and have more offspring (in a way that scales with *f*).

By taking the second derivative $\frac{\partial \Delta \text{IF}}{\partial x^{*}.}$, we find solutions which are local maxima, and hence candidate ESSs, over the relevant parameter space(0 ≤ *R* ≤ 1, 0 ≤ *λ* ≤ 1), which we denote $x_{0}^{*}$ (Maynard Smith & Price, 1973; Taylor & Frank, 1996; Otto & Day, 2007; Lehmann & Rousset, 2014; Biernaskie & West, 2015):

(3) $$x_{0}^{*}=\frac{R\left[f\left(1-\lambda \right)+s\right]}{R\left[f\left(1-\lambda \right)+s-1\right]+1},$$

**Figure 1:**
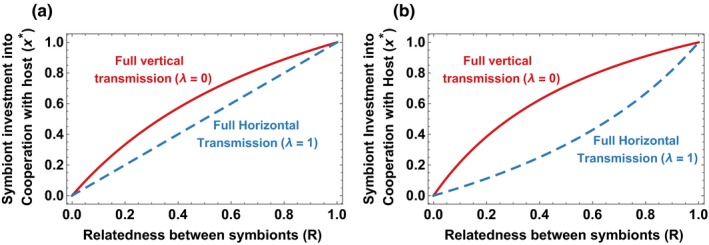
Both transmission mode and relatedness influenced the final level of cooperation that emerged (Equation 3). In (a), host survival and fecundity both increase in the same way with symbiont cooperation (s = f = 1). In (b), host fecundity increases more quickly with symbiont cooperation than host survival does (*s* = 0.5, *f* = 2)

We found that both relatedness and transmission mode influenced the final level of cooperation in this model (Figure 1). Relatedness increases cooperation because it increases the extent to which the benefits of cooperation go to genetic relatives of the actor. This is reflected by an increased weighting of the second term in Equation 2b, resulting in a higher level of cooperation (x*) when fitness is at equilibrium. Vertical transmission increases cooperation because higher levels of vertical transmission increase the extent to which host fecundity can benefit symbionts (Equation 1). This is reflected in Equation 2b by the fact that vertical transmission (lower *λ*) increases the *f*(1–*λ*) component of the group symbiont benefit (second term of Equation 2b). These findings are consistent with previous work that looked just at transmission mode or just at relatedness (Yamamura, 1993; Frank, 1994).

### 2.3 Transmission or relatedness: open model

At this stage, we are interested in asking two different questions of our model. The first question is whether relatedness or transmission mode plays the larger role in determining cooperation. To answer this question, we keep relatedness as an open parameter in our model, allowing us to examine the separate causal influences of relatedness (R) and transmission mode (*λ*). However, in reality, these factors are not independent, since transmission mode can determine relatedness (Taylor, 1992; Frank, 1996; Cooper *et al.*, 2018 ). We can capture this by “closing” the model and expressing relatedness in terms of demographic parameters. Closing the model allows us to ask our second question of why transmission mode influences cooperation: primarily through its direct influence on cooperation per se, or primarily through its influence on relatedness?

To start with, we keep relatedness as an open parameter. Both relatedness and transmission mode influence the equilibrium level of cooperation (Equation 3). For the parameters chosen in Figure 1, it appears that relatedness plays a larger role than transmission mode, in the sense that small changes to relatedness influence the equilibrium level of cooperation more than small changes in transmission mode do (Figure 1). To extend this comparison over all of the potential parameter space, we compared the marginal effect of changes in transmission mode (*λ*) or relatedness (*R*) on the equilibrium level of cooperation.

We calculated the marginal effects by taking the differential of the equilibrium level of cooperation with respect to either relatedness ($\frac{\partial x_{0}^{*}}{\partial R}$) or transmission mode ($\frac{\partial x_{0}^{*}}{\partial \lambda }$). The first of these differentials ($\frac{\partial x_{0}^{*}}{\partial R}$reflects the alignment of fitness interests between symbionts within a host—to what extent should more highly related groups of symbionts cooperate more? The second of these differentials ($\frac{\partial x_{0}^{*}}{\partial \lambda }$) reflects the alignment of fitness interests between a host and its symbionts—to what extent does increased vertical transmission favour a host's symbionts to cooperate more? By comparing the value of the two differentials, we can determine whether relatedness $\left(\left|\frac{\partial x_{0}^{*}}{\partial R}\right|>\left|\frac{\partial x_{0}^{*}}{\partial \lambda }\right|\right)$ or transmission mode $\left(\left|\frac{\partial x_{0}^{*}}{\partial R}\right|<\left|\frac{\partial x_{0}^{*}}{\partial \lambda }\right|\right)$ has a larger influence on the equilibrium level of cooperation.

**Figure 2:**
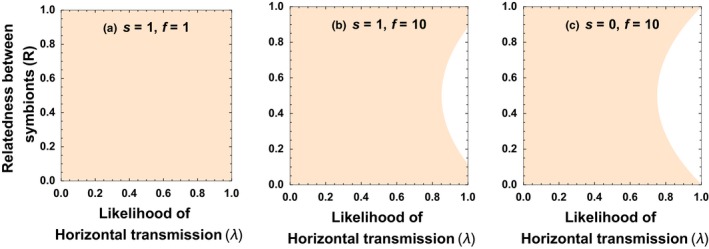
In the first analytical model (Equation 3), relatedness (*R*) usually had a larger influence on the final level of cooperation than transmission mode (*λ*) did. In the orange regions plotted, relatedness had a larger influence than transmission mode, whereas in the white regions, transmission mode had a larger influence than relatedness. Transmission mode only had a larger influence when transmission was mostly horizontal (*λ* > 0.75) and when host fecundity increased more rapidly with symbiont cooperation than host survival did (f>>s)

In Appendix 1, we show that, for most of the possible parameter space, relatedness (*R*) plays a bigger role than transmission mode (*λ*) in determining the final level of cooperation (Figure 2). Specifically, transmission mode only plays a larger role if three conditions are all met: (a) horizontal transmission dominates (*λ* > 0.75); (b) host fecundity accelerates substantially faster with symbiont cooperation than host survival (*f* > 4s); and (c) relatedness is neither maximal nor minimal (0 < *R* < 1; Figure 2).

### 2.4 Transmission and relatedness: closed model

Our next step is to “close” the model by expressing relatedness in terms of demographic parameters (Cooper *et al.*, 2018). We assume that hosts infected by symbionts horizontally are infected by *k_h_* symbionts and that vertically infected hosts are infected by *k_v_* symbionts. In Appendix 2, we show that whole‐group relatedness can now be expressed as:

(4) $$R=\frac{k_{h}\left(1-\lambda \right)+\lambda k_{v}}{k_{h}\left[1+\left(k_{v}-1\right)\lambda \right]},$$

where *k_h_* and *k_v_* give the horizontal and vertical bottleneck sizes, respectively, and *λ* gives the fraction of host offspring that are infected horizontally.

Relatedness depends on the extent to which transmission is vertical or horizontal. Under full horizontal transmission (*λ *= 1), Equation 4 simplifies to $\frac{1}{k_{h}}$, whereas under full vertical transmission, Equation 4 simplifies to 1 (full relatedness). This occurs because horizontal transmission “resets” relatedness by enforcing complete mixing of unrelated symbionts, whereas vertical transmission allows relatedness to increase each generation, since symbionts interact only within a local group.

Next, we further simplify Equation 4 by assuming that horizontally and vertically transmitting symbionts experience the same bottleneck size (*k_h_* = *k_v_* =k) to arrive at:

(5) $$R=\frac{1}{1+\lambda \left(k-1\right)}.$$

By substituting our expression for relatedness (Equation 5) into our expression for the equilibrium level of cooperation ($x_{0}^{*}$; Equation 3), we arrive at a new expression for the equilibrium level of cooperation, which we denote $x_{c}^{*}$:

(6) $$x_{c}^{*}=\frac{f\left(1-\lambda \right)+s}{f\left(1-\lambda \right)+s+\left(k-1\right)\lambda }.$$

**Figure 3:**
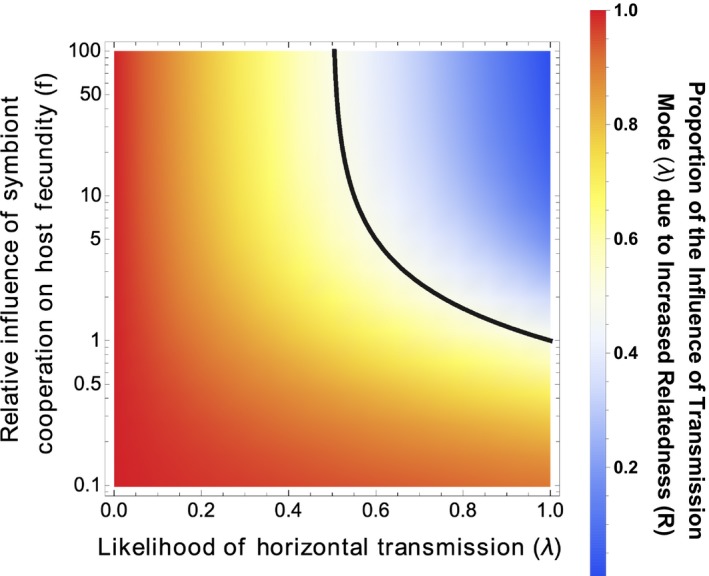
In the second analytical model (Equation 3), transmission mode influenced cooperation primarily through its influence on relatedness. Transmission mode always influenced cooperation more via *R* when transmission was mostly vertical (*λ*  < 0.5) or when host survival increased with symbiont cooperation more quickly than host fecundity did (*f* < s). The dark line plots the point at which transmission mode influences cooperation equally through both routes. For this plot, *s* = 1

We then compared the extent to which transmission mode influences cooperation via its direct influence and via its influence on relatedness. To do this, we first calculated, as before, the marginal effect of changes in transmission mode on the equilibrium level of cooperation for the model with relatedness left open ($\frac{\partial x_{0}^{*}}{\partial \lambda }$). Then, we calculated the total effect of changes in transmission mode on the equilibrium level of cooperation, by taking the differential of the expression for equilibrium cooperation after the model has been closed ($\frac{\partial x_{c}^{*}}{\partial \lambda }$). These two partial derivatives represent, respectively, the influence of transmission mode via its direct influence and the total influence of transmission mode via both influences. We isolate the effect of transmission mode via its influence on *R* by subtracting the first partial derivative ($\frac{\partial x_{0}^{*}}{\partial \lambda }$) from the second ($\frac{\partial x_{c}^{*}}{\partial \lambda }$). By comparing these derivatives, we can then test whether transmission mode matters mostly because it aligns the interests of symbionts sharing a host (by increasing relatedness) or mostly by aligning the interests of symbionts and hosts. In Appendix 3, we show that transmission mode always had a larger influence via its influence on relatedness than via its direct influence $\left(\frac{\frac{\partial x_{c}^{*}}{\partial \lambda }-\frac{\partial x_{0}^{*}}{\partial \lambda }}{\frac{\partial x_{0}^{*}}{\partial \lambda }}>1\right)$, unless: (i) symbiont cooperation increases host fecundity faster than it increases host survival (*f*  > *s*); (ii) and transmission is mostly horizontal (*λ* > 0.5; Figure 3).

Our closed model highlights how focusing just on transmission mode could lead to misleading predictions about the level of cooperation. Equation 5 shows that if transmission is mostly vertical (low *λ*), then relatedness will always be high, because the *λ*(*k*‐1) term will be small. However, if transmission is mostly horizontal (high *λ*), then relatedness can either be high or low, depending on the degree of bottlenecking (the value of *k*) (Equation 5). Consequently, if transmission is mostly horizontal, then focusing just on transmission mode erroneously predicts that a low level of cooperation will evolve, when in fact high levels of cooperation can sometimes evolve (Equation 6).

### 2.5 Simulation

We next wrote an individual‐based simulation in order to check whether our predicted equilibria were evolutionarily stable. Our simulation closely followed our analytical model life cycle (section 2.1), except that we specified the number of hosts and the frequency and size of mutations (Appendix 4). In the simulation, our transmission mode parameter *λ* is the likelihood that each new host receives symbionts horizontally (from the adult host population at large). Correspondingly, 1‐*λ* gives the chance that each host receives symbionts vertically (from its parent).

**Figure 4:**
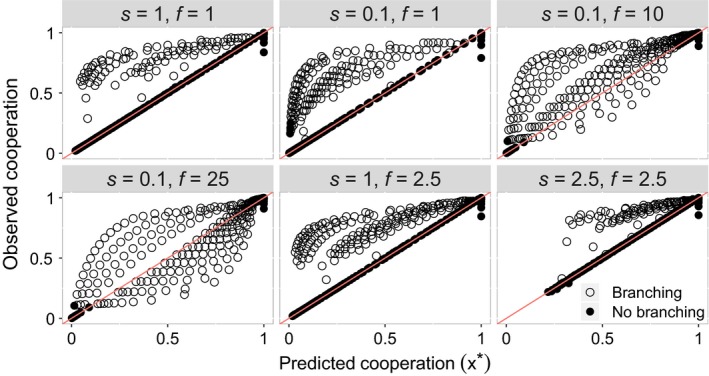
The mean level of cooperation in the symbiont population predicted by our analytical solution (red line) and observed in our simulation (circles). When evolutionary branching did not occur (closed circles), the simulation results closely match the analytical predictions. When evolutionary branching occurred (open circles), the simulation results diverged from the model predictions, and this generally led to a higher level of cooperation than predicted. Predictions are obtained using Equation 6

Our simulation led to two different outcomes. In some simulation runs, the symbiont population remained at our predicted equilibrium level of cooperation, forming a monomorphic population. In these runs, the simulation results closely agreed with the analytical models (Figure 4).

**Figure 5:**
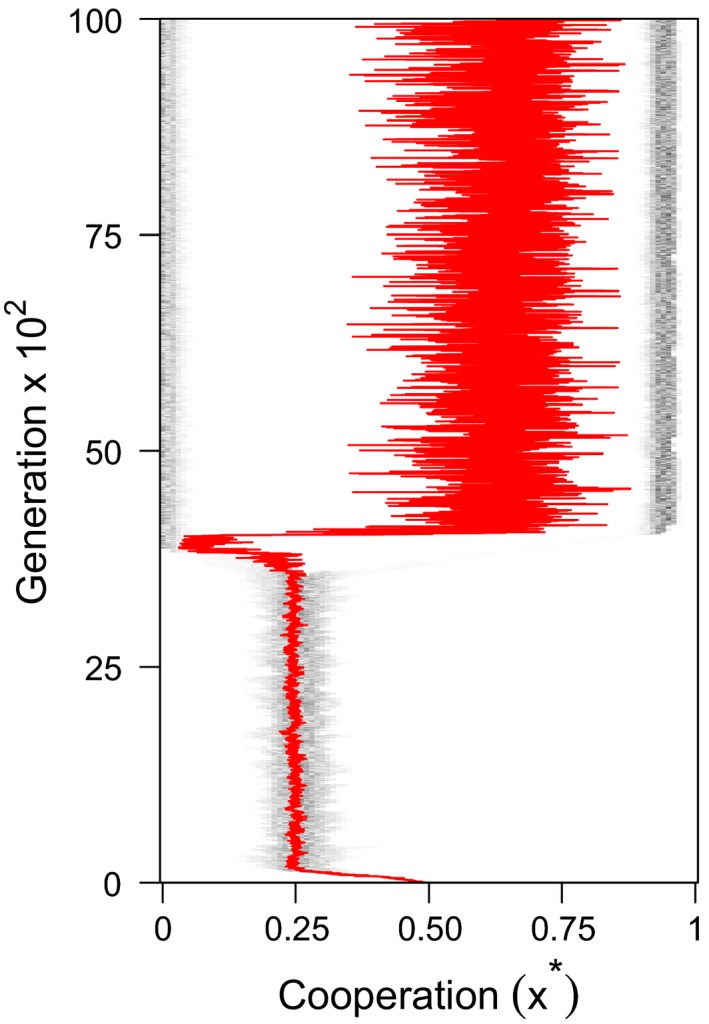
Evolutionary branching. An example simulation where the level of symbiont cooperation branched. In this plot, darker points reflect higher densities of symbionts at each cooperation strategy, and the red line gives the mean level of cooperation in the symbiont population as a whole. The level of cooperation initially approaches a monomorphic value of cooperation predicted by our analytical model at *x* ^*^~0.25, but then, between generations 2,500 and 5,000, it branches to a bimodal equilibrium, with some symbionts cooperating significantly more than others. In this simulation run, following the branching event, the mean level of symbiont cooperation (red line) is significantly higher than it was before. For this simulation run, *s(x) = x* and *f(x) = x*

In other simulation runs, the symbiont population diverged to form a stable polymorphism between strains that cooperated to different degrees (evolutionary branching). In runs when branching occurred, the final mean level of cooperation was usually higher, but occasionally lower, than our predicted equilibrium (Figure 5). In these runs, the final level of cooperation correlated with both transmission mode and relatedness, and it was not possible to disentangle the causal influence of each.

**Figure 6:**
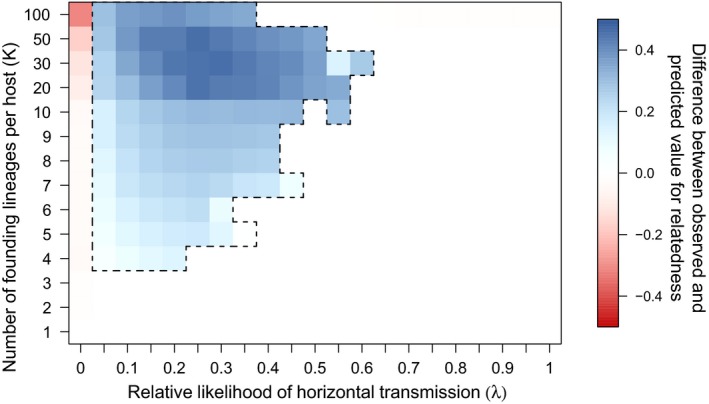
The distribution of evolutionary branching across the simulation. The region of parameter space in which evolutionary branching occurred is outlined by the black dotted line. When evolutionary branching occurred, the observed values of relatedness were significantly higher than the predicted values of relatedness. The red region reflects the fact that when there is no horizontal transmission, and bottlenecking is weak (high *k*), mutation–selection balance can keep noncooperative symbionts in the population transiently (Frank, 1994). For this figure, *s(x) = x* and *f(x) = x*

In the simulation runs when branching occurred, the symbiont population first reached the monomorphic equilibrium predicted by our analytical models, but then diverged to form a stable polymorphism between strains that cooperated to different degrees (Figure 5). In most runs, this resulted in a population of “super‐defectors” that invested the minimum in cooperation. Additionally, in some runs, there were further branching events, leading to more than two populations of symbionts coexisting. When branching occurred, the resulting level of relatedness differed substantially from the relatedness that we predicted based on the demographic parameters (Equation 4; Figure 6). This indicates that in the simulations, unlike in the analytical models, the trait value for cooperation could influence relatedness. We suggest that this may be occur because less cooperative strains are more likely to be in mixed infections than more cooperative strains, since hosts infected only by cooperative strains are more likely to survive than those infected only by noncooperative strains. Consequently, positive feedback could drive more cooperative strains to cooperate more, and less cooperative strains to cooperate less. This feedback cannot occur in the analytical model, because we assume that the symbiont population is at a single equilibrium; however, it can occur in the simulation, where symbionts with very different values for cooperation can interact (Figure 5).

## 3 DISCUSSION

We found, in both our analytical and simulation models, that the relatedness between symbionts in a host was a major determinant of the level at which symbionts cooperate with their hosts (Figures 1, 2 and 4). In contrast, while transmission mode was correlated with the level of symbiont cooperation, this was mainly through its influence on relatedness (Figure 3). Consequently, transmission mode can be a less useful predictor of the level of cooperation, because it is just one of a number of factors that determine relatedness—other factors include the degree of bottlenecking that occurs when symbionts infect new hosts.

Both experimental and across species comparative studies have suggested vertical transmission leads to symbionts that provide greater benefits to hosts (Sachs & Wilcox, 2006; Fisher *et al.*, 2017). Analogous patterns have been found in many parasitic systems, where vertical transmission commonly leads to reduced virulence in both experimental and comparative studies (Bull *et al.*, 1991; Herre, 1993; Messenger *et al.*, 1999; Stewart et al., 2005; Lambrechts & Scott, 2009). Our results suggest that the influence of transmission mode is primarily because of its influence on the relatedness between symbionts sharing a host (Figures 2 and 3). Although we have not modelled every possible scenario, and different life‐history assumptions could lead to different results, we deliberately kept our model simple in order to focus on mechanisms which are likely to be of widespread importance, such as within‐host competition for resources. Consequently, we expect our conclusions to be widely applicable (Herre, 1993; Frank, 1996; West & Buckling, 2003; Alizon et al., 2013; Speare *et al.*, 2018).

We found that evolutionary branching occurred across much of the parameter space in our simulations, leading to stable coexistence between two strains, which cooperate to different degrees (Figure 5). Evolutionary branching has been observed in game theory models in which there are saturating benefit and cost functions near the equilibrium, or where cooperation is linked with another trait under evolution, such as dispersal, as well as in models of parasite virulence (Nowak & May, 1994; Doebeli et al., 2004; El Mouden & Gardner, 2008; Wakano & Lehmann, 2014; Mullon, Keller, & Lehmann, 2016, 2018). Evolutionary branching has also been observed in the early stages of experimentally evolved mutualisms, resulting in variation in the extent to which members of one species cooperate with the other (Harcombe *et al.*, 2018). However, it is unclear whether this variation is likely to be sustained over evolutionary time periods, leading to variance in symbiont partner quality, or whether this variance will be eroded. This is because variation in the level of cooperation could select for hosts to preferentially reward cooperators and/or sanction noncooperators, as has been observed in a number of mutualisms (Noë & Hammerstein, 1995; Johnstone & Bshary, 2002; West et al., 2002; West *et al.*, 2002; Kiers et al., 2003; Jandér & Herre, 2010; Kiers *et al.*, 2011; Wyatt et al., 2014). The consequence of such rewarding and sanctions would select against less cooperative symbionts, reducing the variance in the level of cooperation (West *et al.*, 2002), which could reduce the likelihood that we observe coexistence in nature. Other explanations for the coexistence of symbionts which cooperate to different degrees include different symbiont genotypes adapted to different hosts (Bever et al., 2009; Gubry‐Rangin et al., 2010; Gordon *et al.*, 2016).

To conclude, our results also emphasize the role of transmission route and relatedness in major evolutionary transitions. We predict that when symbionts are clonal (*R* = 1), they should cooperate at the highest level possible with their hosts (x*=1). In this case, there is no conflict between symbionts, and the interests of the hosts and symbionts can be perfectly aligned with regard to how much the symbionts should cooperate (Bordenstein & Theis, 2015; Moran & Sloan, 2015). An alignment of interests between hosts and symbionts is one of the factors required for a major evolutionary transition to a higher level organism/individual (Maynard Smith & Szathmary, 1995; Gardner & Grafen, 2009; Bourke, 2011; West et al., 2015). Examples of such major transitions include the evolution of the eukaryotic cell, plastid endosymbiosis, and some obligate endosymbionts in insects (West *et al.*, 2015). Our results suggest that vertical transmission, combined with population bottlenecks, leading to clonal populations of symbionts within hosts, could play a key role in driving major transitions involving hosts and their symbionts. Furthermore, this is analogous to how clonality or monogamy can align interests and hence drive major transitions between members of the same species (Boomsma, 2007, 2009; Fisher et al., 2013; West *et al.*, 2015).

## APPENDIX 1

We are interested in whether relatedness (*R*) or transmission mode (*λ*) plays the bigger role in influencing the final level of cooperation in the first analytical model (Equation 3). As a heuristic for this, we determine the effect of marginal changes in either relatedness or transmission mode on the equilibrium level of cooperation. Since the equilibrium level of cooperation depends positively on relatedness, but negatively on increased horizontal transmission, we take the absolute magnitude of the relevant derivatives. Therefore, relatedness plays the bigger role when $\left|\frac{\partial x_{0}^{*}}{\partial R}\right|>\left|\frac{\partial x_{0}^{*}}{\partial \lambda }\right|$. Given the constraints of our model (i.e. *s* > 0, *f* > 0, 0 ≤ *R* ≤ 1, 0 ≤ *λ* ≤ 1), this condition simplifies to $\frac{f\left(1-\lambda \right)+s}{fR\left(1-R\right)}>1$. This holds provided that $\lambda <\lambda^{\prime }$, where $\lambda^{\prime }=\frac{s}{f}+1-R\left(1-R\right)$.

Relatedness always plays the bigger role ($\lambda <\lambda^{\prime }$) when *λ`* > 1, because *λ* is defined between 0 and 1. *λ*` > 1 only when fertility accelerates quickly ($f\geq \frac{s}{R\left(1-R\right)}$), a condition which becomes prohibitively restrictive as *R *→ 0 or *R* → 1, and is least restrictive when *R* = 0.5, at which point f≥4s. Hence, *R* will always play the bigger role provided that fertility accelerates less than four times as quickly as survival (*f* < 4s).

Furthermore, because *λ^`^* is increasing with *s*, we can find the lowest value that *λ^`^* can possibly take, by taking the limit of *λ^`^* as *s *→ 0: $\lim_{s\to 0}\frac{s}{f}+1-R\left(1-R\right)=1-R\left(1-R\right)$. This expression equals 1 whenever *R* is 0 or 1, and is lowest when *R* = 0.5, at which point it evaluates to 0.75. Therefore, relatedness is more important (*λ < λ^`^*) provided transmission is more than 25% vertical (*λ <* 0.75).

To summarize, relatedness (*R*) always plays a bigger role than transmission mode (*λ*) in determining the final level of cooperation, unless the three following conditions are all met: (a) horizontal transmission dominates (*λ >* 0.75); (b) host fecundity accelerates substantially faster with symbiont cooperation than host survival does (*f* < 4*s*); (c) relatedness is intermediate (0 < *R* < 1). We plot the area of parameter space in which relatedness has a larger influence than transmission mode in Figure 2.

## APPENDIX 2

To “close” the model by expressing relatedness in terms of demographic parameters, this we focus on a focal symbiont and calculate the likelihood that a randomly chosen symbiont infecting the same host, with replacement, is identical by descent (Pepper, 2000). We calculate this for both horizontally transmitting symbionts and vertically transmitting symbionts, and then we combine both expressions, weighted by the likelihood that each mode of transmission has occurred. Note that this method assumes that evolution in the focal trait does not influence relatedness.

First, with probability *λ*, all symbionts within the focal symbiont's host were acquired horizontally from a well‐mixed pool of symbionts. Because hosts are infected horizontally by exactly *k_h_* symbiont strains, the probability of sampling the same individual twice is 1/*k_h_*. Because we have assumed a large population of hosts, relatedness to any symbionts which are not identical by descent is zero. Second, with probability 1 – *λ*, all symbionts were inherited vertically from the host's parent. As before, the probability of picking the same individual twice is 1/*k_v_*. With probability (*k_v_* − 1)/*k_v,_* the second symbiont is different than the first. In this case, the probability that both symbionts are identical by descent is given by the relatedness between pairs of symbionts in the parent's host in the previous generation *R*_(_ *_t_*_‐1)_. We therefore derive the relatedness at a given generation *t* (i.e. *R*_(_ *_t_*_)_) of a focal symbiont to a randomly picked partner in the same host (with replacement) as:

(S1) $$R_{\left(t\right)}=\left(1-\lambda \right)\left(\frac{1}{k_{v}}+\frac{k_{v}-1}{k_{v}}R_{\left(t-1\right)}\right)+\lambda \frac{1}{k_{h}}.$$

At equilibrium, relatedness will no longer change from one generation to the next, and thus, solving *R*_(_ *_t_*_)_ = *R*_(_ *_t_*_−1)_ gives the value of relatedness, *R*, at equilibrium:

(S2) $$R=\frac{k_{h}\left(1-\lambda \right)+\lambda k_{v}}{k_{h}\left[1-\left(1-k_{v}\right)\lambda \right]}.$$

Since we assume here that the cooperation trait does not influence relatedness, our expression for relatedness (Equation S2) does not depend on the functional forms of fecundity and survival (*s*(*x*) and *f*(*x*)).

If we assume that symbiont bottleneck sizes are the same for both horizontal and vertical transmission (*k_h_* = *k_v_* =k), then our expression for relatedness (Equation S2) simplifies to

(S3) $$R=\frac{1}{1+\lambda \left(k-1\right)}$$

## APPENDIX 3

Transmission mode can influence the level of cooperation that evolves through two routes: (a) through influencing cooperation per se and (b) through influencing relatedness, which in turn influences cooperation. To capture the first of these routes, we take the differential of cooperation with respect to transmission mode, $\frac{\partial x_{0}^{*}}{\partial \lambda }$. It is difficult to calculate the second route directly, but we instead calculate it indirectly by taking the total effect of transmission mode (which is given by taking $\frac{\partial x_{c}^{*}}{\partial \lambda }$ and substituting the value for R given in Equation S3) and subtracting the effect of transmission per se. Therefore, transmission mode influences cooperation more via its effect on relatedness, than via its effect per se, when $\frac{\left|\frac{\partial x_{c}^{*}}{\partial \lambda }\right|-\left|\frac{\partial x_{0}^{*}}{\partial \lambda }\right|}{\left|\frac{\partial x_{0}^{*}}{\partial \lambda }\right|}>1$. This simplifies to $\frac{f+s}{f\lambda }>2$, which is always true for our constraints (*s* > 0, *f* > 0, 0 ≤ *λ* ≤ 1) provided that $\lambda <\frac{f+s}{2f}$. We know that *λ* ≤ 1, so this condition always holds if $\frac{f+s}{2f}>1$, which is true whenever *f* < *s*.

Furthermore, by taking $\lim_{s\to 0}\frac{f+s}{2f}$, we can also see that this condition always holds provided *λ* ≤ 0.5. Therefore, transmission mode always influences cooperation more via its effect on *R* (indirectly) than via its direct effect per se, if transmission is mostly vertical (*λ *< 0.5) () or when host survival increases with symbiont cooperation more quickly than host fecundity does (*f* < s). In Figure 3, we plot the proportion of the influence of transmission mode that is due to its effect on R; this proportion is given by $\frac{\left|\frac{\partial x_{c}^{*}}{\partial \lambda }\right|-\left|\frac{\partial x_{0}^{*}}{\partial \lambda }\right|}{\left|\frac{\partial x_{c}^{*}}{\partial \lambda }\right|}=1-\frac{f\lambda }{f+s}$.

## APPENDIX 4

We next wrote a simulation in order to relax our analytical assumption of weak selection, based on the life cycle of our analytical model in the main text (section 2.1).

We initialize our simulation with a constant number of hosts *n*. At the start of the simulation, each host is infected by *k* symbionts, with each symbiont's investment into cooperation starting at 0.5. We assume that symbionts reach large population sizes within each host, and so we incorporate mutation by assuming that a constant fraction of the symbiont population (*µ* = 0.001) mutates to the current value for cooperation ± 0.01 (one mutational step up or down). Transmission of symbionts and interactions between symbionts occur as specified in our first analytical model (section 2.1; Equation 1).

We ran the simulation for 10^6^ generations, after determining that this was sufficient to allow evolutionary branching events to occur (Figure 5), by comparing simulation runs for 10^5 and 10^6 generations. We determined whether or not evolutionary branching had occurred by visual inspection and found that we could positively identify branching events when the range of cooperation values (the highest value minus the lowest) in the symbiont population was greater than $0.1+{\overset{¯}{x}}^{2}$, where $\overset{¯}{x}$ is the mean level of cooperation in the symbiont population. We recorded observed whole‐group relatedness in the simulation (Figure 6) by calculating the correlation coefficient between a focal symbiont's trait value for cooperation and the average value for cooperation within its host (Pepper, 2000).

We wrote the simulation in MATLAB version R2016a and obtained results using the University of Oxford Advanced Research Computing (ARC) Facility (Richards, 2015). We analysed the results in R version 3.4 and produced figures with ggplot2 (Wickham et al., 2019). A full simulation life cycle, MATLAB scripts to run the simulation, text files of results produced, and R code with data analysis and figure production are available online at https://osf.io/puwq3/.

## References

[jeb13505-bib-0001] Alizon, S. , de Roode, J. C. , & Michalakis, Y. (2013). Multiple infections and the evolution of virulence. Ecol. Lett., 16, 556–567. 10.1111/ele.12076 23347009

[jeb13505-bib-0002] Bever, J. D. , Richardson, S. C. , Lawrence, B. M. , Holmes, J. , & Watson, M. (2009). Preferential allocation to beneficial symbiont with spatial structure maintains mycorrhizal mutualism. Ecol. Lett., 12, 13–21. 10.1111/j.1461-0248.2008.01254.x 19019195

[jeb13505-bib-0003] Biernaskie, J. M. , & West, S. A. (2015). Cooperation, clumping and the evolution of multicellularity. Proceedings of the Royal Society B: Biological Sciences, 282, 20151075. 10.1098/rspb.2015.1075 PMC4632621 26246549

[jeb13505-bib-0004] Boomsma, J. J. (2007). Kin selection versus sexual selection: Why the ends do not meet. Curr. Biol., 17, R673–683. 10.1016/j.cub.2007.06.033 17714661

[jeb13505-bib-0005] Boomsma, J. (2009). Lifetime monogamy and the evolution of eusociality. Philosophical Transactions of the Royal Society B: Biological Sciences, 364, 3191–3207. 10.1098/rstb.2009.0101 PMC2781870 19805427

[jeb13505-bib-0006] Bordenstein, S. R. , & Theis, K. R. (2015). Host Biology in Light of the Microbiome: Ten Principles of Holobionts and Hologenomes. PLoS Biol., 13, e1002226 10.1371/journal.pbio.1002226 26284777 PMC4540581

[jeb13505-bib-0007] Bourke, A. F. G. (2011). Principles of social evolution. Oxford, New York: Oxford University Press.

[jeb13505-bib-0008] Buchner, P. (1965). Endosymbiosis of animals with plant microorganisms , Revised ed New York, NY: Interscience Publishers / John Wiley.

[jeb13505-bib-0009] Bull, J. J. , & Molineux, I. J. (1992). Molecular Genetics of Adaptation in an Experimental Model of Cooperation. Evolution, 46, 882–895. 10.1111/j.1558-5646.1992.tb00606.x 28564403

[jeb13505-bib-0010] Bull, J. J. , Molineux, I. J. , & Rice, W. R. (1991). Selection of benevolence in a host‐parasite system. Evolution, 45(4), 875–882. 10.1111/j.1558-5646.1991.tb04356.x 28564051

[jeb13505-bib-0011] Cooper, G. A. , Levin, S. R. , Wild, G. , & West, S. A. (2018). Modeling relatedness and demography in social evolution. Evolution Letters, 2, 260–271. 10.1002/evl3.69

[jeb13505-bib-0012] Doebeli, M. , Hauert, C. , & Killingback, T. (2004). The evolutionary origin of cooperators and defectors. Science, 306, 859–862. 10.1126/science.1101456 15514155

[jeb13505-bib-0013] Douglas, A. E. (1998). Nutritional Interactions in Insect‐Microbial Symbioses: Aphids and Their Symbiotic Bacteria Buchnera. Annual Review Entomology, 43, 17–37. 10.1146/annurev.ento.43.1.17 15012383

[jeb13505-bib-0014] Ewald, P. W. (1987). Transmission modes and evolution of the parasitism‐mutualism continuuma. Ann. N. Y. Acad. Sci., 503, 295–306. 10.1111/j.1749-6632.1987.tb40616.x 3304078

[jeb13505-bib-0015] Ferdy, J. , & Godelle, B. (2005). Diversification of transmission modes and the evolution of mutualism. Am. Nat., 166, 613–627. 10.1086/491799 16224726

[jeb13505-bib-0016] Fisher, R. M. , Cornwallis, C. K. , & West, S. A. (2013). Group formation, relatedness, and the evolution of multicellularity. Curr. Biol., 23, 1120–1125. 23746639 10.1016/j.cub.2013.05.004

[jeb13505-bib-0017] Fisher, R. M. , Henry, L. M. , Cornwallis, C. K. , Kiers, E. T. , & West, S. A. (2017). The evolution of host‐symbiont dependence. Nat. Commun., 8 10.1038/ncomms15973 PMC5500886 28675159

[jeb13505-bib-0018] Foster, K. R. , & Wenseleers, T. (2006). A general model for the evolution of mutualisms. J. Evol. Biol., 19, 1283–1293. 10.1111/j.1420-9101.2005.01073.x 16780529

[jeb13505-bib-0019] Frank, S. A. (1994). Kin selection and virulence in the evolution of protocells and parasites. Proceedings of the Royal Society B: Biological Sciences, 258, 153–161. 10.1098/rspb.1994.0156 7838853

[jeb13505-bib-0020] Frank, S. A. (1996). Models of parasite virulence. Q. Rev. Biol., 71, 37–78. 10.1086/419267 8919665

[jeb13505-bib-0021] Frank, S. A. (2010). A general model of the public goods dilemma. J. Evol. Biol., 23, 1245–1250. 10.1111/j.1420-9101.2010.01986.x 20345809 PMC2903212

[jeb13505-bib-0022] Gardner, A. , & Grafen, A. (2009). Capturing the superorganism: A formal theory of group adaptation. J. Evol. Biol., 22, 659–671. 10.1111/j.1420-9101.2008.01681.x 19210588

[jeb13505-bib-0023] Gordon, B. R. , Klinger, C. R. , Weese, D. J. , Lau, J. A. , Burke, P. V. , Dentinger, B. T. M. , & et al (2016). Decoupled genomic elements and the evolution of partner quality in nitrogen‐fixing rhizobia. Ecol. Evol., 6, 1317–1327. 10.1002/ece3.1953 27087920 PMC4775534

[jeb13505-bib-0024] Gubry‐Rangin, C. , Garcia, M. , & Béna, G. (2010). Partner choice in Medicago Truncatula‐Sinorhizobium symbiosis. Proceedings of the Royal Society B: Biological Sciences, 277, 1947–1951. 10.1098/rspb.2009.2072 PMC2880089 20200033

[jeb13505-bib-0025] Hamilton, W. D. (1964). The genetical evolution of social behaviour. I. J. Theor. Biol., 7, 1–16. 10.1016/0022-5193(64)90038-4 5875341

[jeb13505-bib-0026] Harcombe, W. R. , Chacón, J. M. , Adamowicz, E. M. , Chubiz, L. M. , & Marx, C. J. (2018). Evolution of bidirectional costly mutualism from byproduct consumption. Proceedings of the National Academy of Sciences USA, 115(47), 12000–12004. 10.1073/pnas.1810949115 PMC6255176 30348787

[jeb13505-bib-0027] Harrower, M. , & Brewer, C. A. (2003). ColorBrewer.org: An online tool for selecting colour schemes for maps. The Cartographic Journal, 40, 27–37. 10.1179/000870403235002042

[jeb13505-bib-0028] Herre, E. A. (1993). Population structure and the evolution of virulence in nematode parasites of fig wasps. Science, 259, 1442–1445. 10.1126/science.259.5100.1442 17801279

[jeb13505-bib-0029] Herre, E. A. (1995). Factors affecting the evolution of virulence: Nematode parasites of fig wasps as a case study. Parasitology, 111, S179–S191. 10.1017/S0031182000075880

[jeb13505-bib-0030] Herre, E. A. , Knowlton, N. , Mueller, U. G. , & Rehner, S. A. (1999). The evolution of mutualisms: Exploring the paths between conflict and cooperation. Trends Ecol. Evol., 14, 49–53. 10.1016/S0169-5347(98)01529-8 10234251

[jeb13505-bib-0031] Jandér, K. C. , & Herre, E. A. (2010). Host sanctions and pollinator cheating in the fig tree–fig wasp mutualism. Proceedings of the Royal Society B: Biological Sciences, 277, 1481–1488. 10.1098/rspb.2009.2157 PMC2871839 20071379

[jeb13505-bib-0032] Johnstone, R. A. , & Bshary, R. (2002). From parasitism to mutualism: Partner control in asymmetric interactions. Ecol. Lett., 5, 634–639. 10.1046/j.1461-0248.2002.00358.x

[jeb13505-bib-0033] Karakashian, S. J. (1963). Growth of paramecium bursaria as influenced by the presence of algal symbionts. Physiol. Zool., 36, 52–68. 10.1086/physzool.36.1.30152738

[jeb13505-bib-0034] Kiers, E. T. , Rousseau, R. A. , West, S. A. , & Denison, R. F. (2003). Host sanctions and the legume–rhizobium mutualism. Nature, 425, 78–81. 10.1038/nature01931 12955144

[jeb13505-bib-0035] Kiers, E. T. , Duhamel, M. , Beesetty, Y. , Mensah, J. A. , Franken, O. , Verbruggen, E. ,et al (2011). Reciprocal rewards stabilize cooperation in the Mycorrhizal symbiosis. Science, 333, 880–882. 10.1126/science.1208473 21836016

[jeb13505-bib-0036] Lambrechts, L. , & Scott, T. W. (2009). Mode of transmission and the evolution of arbovirus virulence in mosquito vectors. Proceedings of the Royal Society B: Biological Sciences, 276, 1369–1378. 10.1098/rspb.2008.1709 PMC2660968 19141420

[jeb13505-bib-0037] Lehmann, L. , & Rousset, F. (2014). The genetical theory of social behaviour. Philosophical Transactions of the Royal Society B: Biological Sciences, 369, 20130357 10.1098/rstb.2013.0357 PMC3982659 24686929

[jeb13505-bib-0038] Lowe, C. D. , Minter, E. J. , Cameron, D. D. , & Brockhurst, M. A. (2016). Shining a light on exploitative host control in a photosynthetic endosymbiosis. Curr. Biol., 26, 207–211. 10.1016/j.cub.2015.11.052 26748854

[jeb13505-bib-0039] Maynard Smith, J. , & Price, G. R. (1973). The logic of animal conflict. Nature, 246, 15–18. 10.1038/246015a0

[jeb13505-bib-0040] Maynard Smith, J. , & Szathmary, E. (1995). The major transitions in evolution. New York, NY: Oxford University Press.

[jeb13505-bib-0041] Messenger, S. L. , Molineux, I. J. , & Bull, J. J. (1999). Virulence evolution in a virus obeys a trade‐off. Proc. R. Soc. Lond. B Biol. Sci., 266, 397–404. 10.1098/rspb.1999.0651 PMC1689683 10097397

[jeb13505-bib-0042] Moran, N. A. , & Sloan, D. B. (2015). The hologenome concept: Helpful or hollow? PLoS Biol., 13, e1002311 10.1371/journal.pbio.1002311 26636661 PMC4670207

[jeb13505-bib-0043] El Mouden, C. , & Gardner, A. (2008). Nice natives and mean migrants: The evolution of dispersal‐dependent social behaviour in viscous populations. J. Evol. Biol., 21, 1480–1491. 10.1111/j.1420-9101.2008.01614.x 18811663

[jeb13505-bib-0044] Mullon, C. , Keller, L. , & Lehmann, L. (2016). Evolutionary stability of jointly evolving traits in subdivided populations. Am. Nat., 188, 175–195. 10.1086/686900 27420783

[jeb13505-bib-0045] Mullon, C. , Keller, L. , & Lehmann, L. (2018). Social polymorphism is favoured by the co‐evolution of dispersal with social behaviour. Nature Ecology & Evolution, 2, 132 10.1038/s41559-017-0397-y 29203923

[jeb13505-bib-0046] Noë, R. , & Hammerstein, P. (1995). Biological markets. Trends Ecol. Evol., 10, 336–339. 10.1016/S0169-5347(00)89123-5 21237061

[jeb13505-bib-0047] Nowak, M. A. , & May, R. M. (1994). Superinfection and the evolution of parasite virulence. Proc. R. Soc. Lond. B Biol. Sci., 255, 81–89. 10.1098/rspb.1994.0012 8153140

[jeb13505-bib-0048] Otto, S. P. , & Day, T. (2007). A biologist’s guide to mathematical modeling in ecology and evolution. Princeton, NJ: Princeton University Press.

[jeb13505-bib-0049] Pepper, J. W. (2000). Relatedness in trait group models of social evolution. J. Theor. Biol., 206, 355–368. 10.1006/jtbi.2000.2132 10988021

[jeb13505-bib-0050] Richards, A. (2015). University of Oxford advanced research computing. Zenodo. 10.5281/zenodo.22558

[jeb13505-bib-0051] Sachs, J. L. , & Wilcox, T. P. (2006). A shift to parasitism in the jellyfish symbiont Symbiodinium microadriaticum. Proc. R. Soc. Lond. B Biol. Sci., 273, 425–429. 10.1098/rspb.2005.3346 PMC1560209 16615208

[jeb13505-bib-0052] Speare, L. , Cecere, A. G. , Guckes, K. R. , Smith, S. , Wollenberg, M. S. , Mandel, M. J. , et al (2018). Bacterial symbionts use a type VI secretion system to eliminate competitors in their natural host. Proceedings of the National Academy of Sciences USA, 115(36), E8528–E8537. 10.1073/pnas.1808302115 PMC6130350 30127013

[jeb13505-bib-0053] Stewart, A. D. , Logsdon, J. M. , & Kelley, S. E. (2005). An empirical study of the evolution of virulence under both horizontal and vertical transmission. Evolution, 59, 730–739. 10.1111/j.0014-3820.2005.tb01749.x 15926685

[jeb13505-bib-0054] Taylor, P. D. (1992). Altruism in viscous populations — an inclusive fitness model. Evol Ecol, 6, 352–356.

[jeb13505-bib-0055] Taylor, P. D. , & Frank, S. A. (1996). How to make a kin selection model. J. Theor. Biol., 180, 27–37. 10.1006/jtbi.1996.0075 8763356

[jeb13505-bib-0056] Wakano, J. Y. , & Lehmann, L. (2014). Evolutionary branching in deme‐structured populations. J. Theor. Biol., 351, 83–95. 10.1016/j.jtbi.2014.02.036 24631046

[jeb13505-bib-0057] Wang, L. (2016). make use of color palettes from http://colorbrewer2.org in Mathematica: https://github.com/wanglongqi/ColorBrewer

[jeb13505-bib-0058] West, S. A. , & Buckling, A. (2003). Cooperation, virulence and siderophore production in bacterial parasites. Proc. R. Soc. Lond. B Biol. Sci., 270, 37–44. 10.1098/rspb.2002.2209 PMC1691207 12590769

[jeb13505-bib-0059] West, S. A. , Kiers, E. T. , Pen, I. , & Denison, R. F. (2002). Sanctions and mutualism stability: When should less beneficial mutualists be tolerated? J. Evol. Biol., 15, 830–837. 10.1046/j.1420-9101.2002.00441.x

[jeb13505-bib-0060] West, S. A. , Kiers, E. T. , Simms, E. L. , & Denison, R. F. (2002). Sanctions and mutualism stability: Why do rhizobia fix nitrogen? Proc. R. Soc. Lond. B Biol. Sci., 269, 685–694. 10.1098/rspb.2001.1878 PMC1690951 11934359

[jeb13505-bib-0061] West, S. A. , Fisher, R. M. , Gardner, A. , & Kiers, E. T. (2015). Major evolutionary transitions in individuality. Proceedings of the National Academy of Sciences USA, 112, 10112–10119. 10.1073/pnas.1421402112 PMC4547252 25964342

[jeb13505-bib-0062] Wickham, H. , Chang, W. , Henry, L. , Pedersen, T.L. , Takahashi, K. , Wilke, C. , et al. (2019). ggplot2: Create elegant data visualisations using the grammar of graphics.

[jeb13505-bib-0063] Wyatt, G. A. K. , Kiers, E. T. , Gardner, A. , & West, S. A. (2014). A biological market analysis of the plant‐mycorrhizal symbiosis. Evolution, 68, 2603–2618. 10.1111/evo.12466 24909843

[jeb13505-bib-0064] Yamamura, N. (1993). Vertical transmission and evolution of mutualism from parasitism. Theor. Popul. Biol., 44, 95–109. 10.1006/tpbi.1993.1020

[jeb13505-bib-0065] Yamamura, N. (1996). Evolution of mutualistic symbiosis: A differential equation model. Researches on Population Ecology, 38, 211–218. 10.1007/BF02515729

